# Pulmonary Emphysema: Current Understanding of Disease Pathogenesis and Therapeutic Approaches

**DOI:** 10.3390/biomedicines13092120

**Published:** 2025-08-30

**Authors:** Abderrazzak Bentaher, Olivier Glehen, Ghania Degobert

**Affiliations:** 1 Inflammation and Immunity of the Respiratory Epithelium, INSERM, Claude Bernard Lyon 1 University, UR3738, South Medical University Hospital, 165 Chemin du Petit Revoyet, 69395 Oullins-Pierre-Benite, France; 2 Claude Bernard Lyon 1 University, CICLY-UR3738, South Medical University Hospital, 165 Chemin du Grand Revoyet, 69395 Pierre-Benite, France; olivier.glehen@chu-lyon.fr; 3 LAGEPP—UMR 5007, CNRS, University of Lyon, Claude Bernard Lyon 1 University, 69622 Villeurbanne, France; ghania.hamdi-degobert@univ-lyon1.fr

**Keywords:** emphysema, pathogenic mechanisms, animal experimental models, available therapeutics, exploratory therapies

## Abstract

Pulmonary emphysema, the main component of chronic obstructive pulmonary disease, is a chronic lung inflammatory disease characterized by the loss of lung elasticity and impaired gas exchange due in large part to the destruction of alveolar walls. Cigarette smoking represents the most frequent etiologic factor, but other factors involving environmental pollution and respiratory infections contribute to disease pathogenesis and worsening. In this review, we provide a review about emphysema covering risk factors; underlying mechanisms of disease pathogenesis; experimental models that mimic, as closely as possible, human disease features; and available therapeutics. Lastly, exploratory therapeutic approaches aimed at improving patient health through evidence-based and personalized medicine are presented as well.

## 1. Pulmonary Emphysema

Chronic obstructive pulmonary disease (COPD) is an “umbrella” term encompassing lung diseases that hamper/worsen gradually lung airflow and ultimately proper breathing [1]. One of the most common COPD components is emphysema. This latter is defined as the gradual enlargement of distal air spaces beyond the terminal bronchioles caused by the destruction of the walls of these structures [2]. To picture the disease development, air sacs rupture over time and form one “big pouch” where air is trapped. Consequently, not only does oxygen move hardly into systemic circulation but also the lungs slowly “overfill”. As such, while early on, the symptoms may be mild and overlooked, they become more noticeable and significantly impact human life quality as the disease progresses. Indeed, this translates in shortness of breath, chronic cough with mucus production, wheezing, and chest tightness. Other symptoms may include weight loss, fatigue, anxiety, depression, cardiovascular problems, and sleep deprivation. Patients with emphysema are also at higher risk of developing respiratory infections that exacerbate the pathology. Of importance, accurate diagnosis is important to differentiate between emphysema and other respiratory conditions that have similar but confusing symptoms and to ensure the appropriate treatment and management of the disease. Of relevance, this “epidemic” disease is expected to rank as the third cause of mortality in the coming years [3]. Currently, it afflicts approximately 9% to 10% of the population throughout the world, representing a high economic burden [4].

## 2. Risk Factors

The primary risk factor for emphysema development is cigarette smoking (CS). But other relatively important risk factors involve the environment including air pollution, dust, and chemicals [4,5,6,7,8,9]. Regarding air pollution, we have identified and reported recently that exposure to the secondary organic aerosols (SOAs), part of the particulate matter, represents a critical risk factor for emphysema development [10,11]. A genetic condition, namely alpha-1 antitrypsin (main physiologic neutrophil elastase inhibitor) deficiency, has been also documented to increase the risk of developing emphysema, particularly in individuals who smoke [12]. Table 1 summarizes documented risk factors leading to emphysema [4,5,6,7,8,9,10,11,12,13,14]. Of interest, repeated respiratory infections, such as pneumonia, and asthma also precipitate or increase the risk of developing emphysema [13,14].

## 3. Hypotheses of Disease Pathogenesis

**Figure 1:**
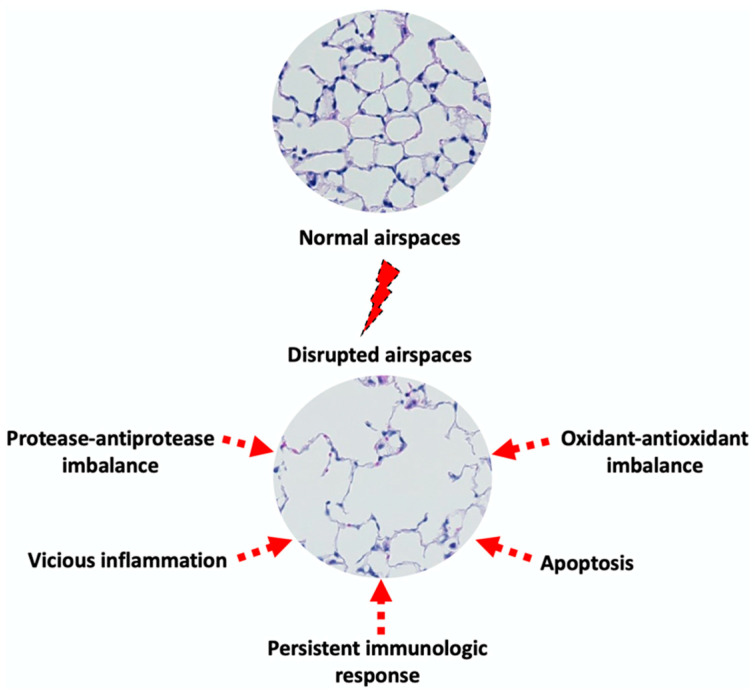
Proposed mechanisms of emphysema pathogenesis.

The pathogenesis of emphysema is complex and not completely understood. Emphysema affects primarily lung alveoli and indirectly small airways. These physiological changes involve damage to the alveolar walls and the loss of elasticity, all of which lead to the enlargement of the air spaces beyond the normal size and collapsed small airways [15]. A series of hypotheses have been proposed to delineate the underlying mechanisms behind these morphological changes (Figure 1).

### 3.1. Protease–Antiprotease Imbalance

According to the protease–antiprotease hypothesis, prolonged exposure to pathogenic factors (e.g., cigarette smoking) induces an abnormal inflammation characterized by the presence of uncontrolled protease-rich milieu. This imbalance between proteases and their physiologic inhibitors “favors” active proteases to degrade lung structural proteins, particularly elastin, leading to the loss of organ architecture, hence emphysema development [16,17]. In support of this, a series of studies reported that gene targeting of defined proteases contributed to the protection of mice against cigarette smoke (CS)-induced emphysema [18,19]. Moreover, the intrapulmonary instillation of proteases in experimental animal models resulted in typical features of this pathologic condition. This was a direct evidence, as mentioned above, that the inherited deficiency of alpha-1 antitrypsin, the primary physiologic inhibitor of the protease neutrophil elastase (NE), predisposes to emphysema [20,21].

### 3.2. Oxidant–Antioxidant Imbalance

Exposure to irritants such as cigarette smoke or air pollution leads to an increase in the levels of exogenous oxidants and oxidative stress in the lungs. Recruited and activated inflammatory cells such as neutrophils, macrophages, and activated epithelial cells represent also a relatively important source for endogenous oxidant production [22]. Interestingly, irritants such as cigarette smoke are known to activate inflammatory cells releasing oxidants and pro-inflammatory cytokines. These latter will lead in turn to the recruitment of more neutrophils and macrophages, perpetuating a vicious cycle of endogenous oxidant production. This oxidative system includes, among others, superoxide anions, hydroxyradicals, hypochlorous acid, hydrogen peroxide, and nitric oxide. These highly reactive species have the capacity to target directly host macromolecules such as proteins, lipids, and nucleic acids resulting in cell dysfunction and/or death (e.g., apoptosis) [23,24]. Studies have also reported that oxidants inactivate protease physiologic inhibitors, thereby amplifying proteolysis-mediated tissue destruction. In principle, the normal production of oxidants is balanced by various host antioxidant enzymes in the lungs. Among these enzymes are catalase, superoxide dismutase, and glutathion (GSH) [25]. Of relevance, antioxidant enzyme synthesis is regulated by nuclear factor erythroid 2-related factor 2 (Nrf2), which translocates to the nucleus and transcribes their corresponding genes [26]. Of relevance, disruption or alteration in the expression of Nrf2 in animal models results in severe emphysema [27]. As a consequence, this causes damage to both the alveolar walls as well as other lung tissues, leading to the development of emphysema.

### 3.3. Vicious Cycle of Inflammation

Normally, the lung is equipped with an appropriate defensive system (e.g., cilia, cough, mucus to expel foreign bodies) that maintains it in a homeostatic state. But inflammation, characterized by the recruitment of inflammatory cells, is recognized as a key process in emphysema pathogenesis from disease initiation and throughout its progression. The hypothesis is that chronic exposure to irritants including cigarette smoke leads to the release of an inflammatory arsenal by host cells ranging from cytokines, oxidants, and proteases that directly or indirectly target host structural molecules and damage the alveolar walls, all of which promote the development of emphysema. The host-derived or newly generated mediators (e.g., elastin peptides) call in more inflammatory cells, perpetuating a vicious inflammatory cycle [28].

With respect to elastin peptides, they are generated following the protease-mediated cleavage of elastin. We and others have reported that these peptides, also called elastokines, exhibit chemotactic and pro-inflammatory properties that could potentially drive the progression of airspace enlargement in a vicious cycle, making them a potential theragnostic target in emphysema [29]. Indeed, they could serve as a disease marker and as an indirect therapeutic by neutralizing them (e.g., monoclonal antibodies, peptide traps) or blocking their interaction with elastin receptor (e.g., synthetic decoy peptides).

### 3.4. Persistent Immunologic Response

Analyses of bronchoalveolar lavages fluids and/or tissues of emphysematous lungs have revealed the predominance of macrophages regardless of the severity degree of the pathology. These cells not only have “somewhat” impaired phagocytic function but also release tissue-damaging molecules [30]. Lymphocytes, especially CD8+ types, contribute to emphysema pathogenesis [28]. In fact, this cell-mediated inflammatory response seems to persist even after smoking cessation [31].

### 3.5. Apoptosis

Among the driving mechanisms for emphysema pathogenesis, a series of studies reported a role for apoptosis (programmed cell death) in the breakdown of alveolar walls. Analyses of human emphysematous lung tissues revealed increased apoptotic epithelial and endothelial cells within the alveolar septa [32]. Similar findings were confirmed in experimental animal models of lung CS exposure and various pro- and anti-apoptotic mediators have been identified [33]. These latter include, for instance, CS, oxidants, ceramide or adenosine, and focal adhesion kinase or vascular endothelial growth factor [34].

### 3.6. Lung Elastic Fiber Injury

Although this mechanism is a consequence, but not a primary cause of disease development, elastic fiber injury plays yet a detrimental role in the initiation and progression of emphysema by different ways, as supported by experimental and/or human studies. Elastic fibers (mainly elastin and associated microfibrils (e.g., fibrillin)) are essential for both the structural integrity of alveolar septa and small airways and tethering of alveoli and bronchioles, ensuring, thus, mechanical recoil during exhalation and preventing alveoli and bronchiole overdistention and collapse, respectively. These structural components can be altered by at least two means. Proteases (e.g., neutrophil elastase, MMP-12, and cathepsins) degrade elastin and other elastic fiber components and cigarette smoke or inflammatory cell-derived oxidants inactivate protease physiologic inhibitors (e.g., A1AT) and/or oxidize elastin directly, all of which lead to elastin fiber breakdown [35,36]. Consequently, the net result of these effects is the hallmark of emphysema, airspace enlargement.

### 3.7. Disruption of Mechanical Forces

Regarding this latter, this is an irreversible process that results in impaired gas exchange. Another parameter that plays a significant role in this process is the disruption of mechanical forces [37,38]. These latter include stretching, recoil, and tension that allow mechanical stability and maintain alveolar wall structure and function. Of importance as well, the signaling mediated by these forces contributes to regulate cell behavior in terms of survival, proliferation, and repair. Such disruption highly likely contributes to airspace enlargement through the above-mentioned mechanisms [39,40]. Moreover, the nonuniform lung tissue damage leads to the generation of “stress concentrations zones” and compensatory overexpansion (by remaining alveoli), both of which lead to additional mechanical strain and perpetuate the cycle of damage and progressive airspace enlargement.

While we describe several mechanisms that underlie lung injury in emphysema, their relative importance and contribution could be prioritized based on both experimental and clinical evidence. Hence, the imbalance of proteases and their physiologic inhibitors is regarded as the most central hypothesis for genetic (alpha-1 antitrypsin deficiency) and triggered (e.g., smoking-induced) emphysema. The relative importance of the other mechanisms could be ranked in descending order as follows. The oxidative stress is more relevant in smokers and individuals with chronic inflammation, knowing that it both amplifies protease activity and increases the apoptosis of structural lung cells, leading to further alveolar wall injury. The chronic recruitment of neutrophils, macrophages, and CD8+ T-cells engenders a “low-grade inflammation” but remains the source of both proteases and oxidants. Although, not an initiating factor, apoptosis contributes to the progression and irreversibility of emphysema through the loss of alveolar epithelial and endothelial cells. Both impaired repair mechanisms and the loss of parenchymal tethering (uneven distribution of mechanical stress) are more consequences than primary causes that contribute to the progression and irreversibility of emphysema.

Although this classification remains “somewhat” subjective, these proposed hypotheses may not be individually or mutually exclusive, and the likelihood that they might interact to develop emphysema cannot be ruled out.

## 4. Clinically Relevant Experimental Animal Models

Because of the limitations/difficulties related to human studies due to in part to ethical concerns, the use of experimental animal models becomes an important alternative to investigate disease pathogenesis. Significantly, experimental animal models better our understanding of the underlying mechanisms of emphysema pathophysiology that might ultimately help in the development of therapeutic strategies. Indeed, a series of models have been developed using different “insulting” agents (Table 2). It must be emphasized that these models mimic “some” of the features of the human disease given the physiologic differences between species. Nonetheless, they remain, once again, valuable for mechanistic studies that pertain to human emphysema. Various animals have been employed as experimental models ranging from the mouse, rat, guinea pig, hamster, rabbit, dog, pig, sheep, and monkey, with mice being the widely used animals since gene targeting technology is well-established in this species.

### 4.1. Cigarette-Smoke-Induced Emphysema

It has been largely documented that about 90% of COPD patients are smokers [55] and one of the most important risk factors for emphysema is smoking [56]. In the nineties, a guinea pig model of emphysema was developed by means of chronic exposure to cigarette smoke [41]. Among the characteristic features of this model are an inflammatory response predominated by macrophages and the rupture of alveoli leading to emphysema development, similar to the human response to cigarette smoking [57]. Since then, this model has provided the foundation for experimental research studies of human emphysema. According to the literature review, there are now at least two animal models of exposure to CS [42]. The first is a model whereby only the nose/head is exposed to CS through a hole in the smoking apparatus [43]. The other model corresponds to a whole-body exposure method in which the animal is placed in a smoking box [44]. Overall, both models have generated emphysema-like changes in the lungs, similar to what is seen in human patients. Obviously, data obtained from either model and their interpretations take into consideration the length of CS exposure; the kind of cigarettes; smoke density, duration, and frequency; and species differences and their ages. Also, one has to keep in mind lung anatomical differences between species. Last but not least, the impact of smoking on emphysema development may be influenced by parameters involving pre-existing lung disease, genetics, and diet that are difficult to check for in animal studies.

### 4.2. Elastase-Induced Emphysema

A number of elastase-induced animal models of emphysema have been developed to investigate the pathogenesis of this disease and to assess potential treatments. Commonly used elastases include papain, pig pancreatic elastase (PPE), and human neutrophil elastase (HNE) [45,46], and various animal species have been employed as experimental models, ranging from mice, rats, hamsters, and guinea pigs. These elastases have the capacity to break down proteins, particularly elastic fibers within lung tissues resulting in the destruction of alveolar walls. In addition, they induce a myriad of inflammatory factors that precipitate the rupture of alveolar spaces [47]. Altogether, these elastase-mediated processes lead to the development of an emphysema-like phenotype. Empirically, the elastase is instilled in animal lungs at doses and frequencies that correlate with the severity of lung tissue destruction, hence emphysema. Although this model reproduces changes that are observed in human disease, it does not fully replicate the inflammatory and immune responses that occur in human emphysema. Moreover, the lungs are acutely injured (and emphysema develops rapidly) in these models whereas, in humans, the disease is chronic and progresses over time.

### 4.3. Chemical-Induced Emphysema

Hazardous substances from either natural or man-made origin pollute the air and are constantly inhaled by humans. As such, these pollutants interfere with the human biologic functions, affecting different organs including the lungs and causing respiratory diseases like emphysema (this review). The relationship between these pollutants and lung effects has been investigated using various chemicals. The models involve exposing animals to chemicals such as ozone, nitrogen dioxide, or sulfur dioxide through inhalation [48,49,50]. Thus, exposure to high levels of ozone, a reactive gas of urban smog, has been reported to cause lung injury and emphysema-like changes including airway inflammation and the destruction of the alveoli. Similar observations have been made with nitrogen dioxide and sulfur dioxide, toxic gases that are released from vehicle exhaust and industrial processes, whereby exposure to these chemicals was accompanied with increased inflammation and alveolar damage, typical characteristic features of emphysema [51]. One advantage of chemical-induced emphysema models is that they allow us to study the effects of specific toxic on the development of emphysema. In this regard, secondary organic aerosols (SOAs) emerged as predominant components of atmospheric pollutants. And recently, we showed that the chronic exposure of mice to SOAs resulted in lung inflammation and tissue destruction, as seen in emphysema [10,11]. But these models also have their own limitations, such as the fact that the concentrations and duration of exposure may not represent real-world exposures in humans. In addition, and as mentioned above, the effects of chemical exposure on emphysema may be contingent on factors such as genetics, age, and pre-existing lung disease that are difficult to control in animal studies.

### 4.4. Animal Model of Emphysema Exacerbation

Patients with emphysema frequently undergo episodes of worsened symptoms, resulting, often, in substantial morbidity and mortality [58]. These episodes are referred to as exacerbations and are characterized by increased airway and systemic inflammation leading to decreased lung function. They are triggered mainly by respiratory microbes, which infect lower airways. Animal models have been developed to mimic these human clinical features and investigate their pathophysiology and to test potential therapeutic interventions. A commonly used animal experimental model involves exposing mice or rats to a combination of cigarette smoke and bacterial or viral infections [52]. For example, animals are exposed to cigarette smoke for several weeks to induce chronic airway inflammation and periodically infected (during CS exposure) with bacteria, viruses, fungi to induce an exacerbation phase. Thus, our group and others used the frequently isolated strain, *non-typeable Haemophilus influenzae*, in these episodic exacerbations to set up a mouse model of emphysema exacerbation that was indeed marked by an increased inflammatory response and worsened lung injury [53,54]. As with emphysema models, other animal models of emphysema exacerbation involved genetic manipulation to generate animals with specifically altered genes that have been suspected in the development of this condition.

### 4.5. Gene-Alteration-Induced Emphysema

Advances in both molecular biology technology and the Human Genome Project have greatly helped the development of research projects investigating the connection between disease genesis and genes of interest. Hence, suspected human genes have been targeted in experimental animal models. This could be achieved by various techniques manipulating defined genes (e.g., knock-out, knock-in, RNA interference (RNAi), and, recently, CRISPR-cas9). With respect to emphysema, experimental models of genetically engineered mice have been developed to study the role of specific genes. For example, transgenic mice that overexpress the gene for matrix metalloproteinase-12, an enzyme involved in the breakdown of lung tissue, have been shown to develop emphysema-like changes in the lungs [59]. Knock-out mice that lack genes for neutrophil elastase or its main physiologic inhibitor alpha-1 antitrypsin have also been used as models for emphysema [19,60]. Other genes that have been targeted in animal models of emphysema include those that regulate inflammation, oxidative stress, and lung repair mechanisms (Table 3) [19,27,35,60,61,62,63,64,65,66,67,68,69,70,71,72,73,74]. Clearly, this approach offers the advantage of studying the specific role of individual genes in the development and progression of the disease as well as in identifying potential targets for new therapies.

Of relevance, natural variations due to genetic or environmental factors have been identified in animals, rendering them more susceptible to the development of emphysema. For example, some strains of mice have been found to be more susceptible to developing emphysema (e.g., Tit-skin mice, Beige mice, Blotchy mice, Pallid mice [75]) than others and this susceptibility has been linked to specific genetic variations [76]. Interestingly, such animal models of emphysema allow us to study the interaction between genetic and/or environmental factors in the development of the disease. Studies have shown that exposure to cigarette smoke can increase the risk of emphysema in genetically susceptible mice but not in mice that are more resistant to the disease.

In fine, it must be recognized that all these animal experimental models allow us to study the pathogenesis of emphysema in stable or exacerbated phases and the effects of specific genetic changes on disease development and progression and offer the opportunity to assess the potential effects of various therapeutic interventions such as anti-inflammatory agents, antioxidants, and other protective agents. Meanwhile, these models have some limitations. The effects of genetic mutations (natural or specifically targeted) may not fully reflect the complex interactions between genetic and environmental factors that contribute to the development of emphysema in humans. Additionally, the effects of genetic manipulation on emphysema may be impacted by other factors that include age, sex, and pre-existing lung disease that are difficult to control in animal studies. Furthermore, the complex interactions between genetic and environmental factors that contribute to disease exacerbation in humans may be difficult to replicate in animal models. At any rate, while recognizing these limitations is important, these models are still valuable in advancing our understanding of emphysema pathogenesis and the repercussions of therapeutic strategies.

## 5. Therapeutic Strategies

The damage inflicted to lung tissues in emphysema is unfortunately irreversible. Hence, the objectives of treatment for emphysema patients aim to control/improve symptoms, attenuate inflammation, and slow down (or prevent) disease worsening, all of which should help ameliorate the quality of patient life. These strategic alternatives include non-pharmacological and pharmacological therapeutics taking into consideration the severity of the disease and the underlying causes. The former alternative is out of the scope of this review and is therefore briefly described.

### 5.1. Non-Pharmacological Approaches

Non-pharmacological therapy promotes strategies that relieve symptoms and better the life quality of ill patients. These approaches help alleviate symptoms including shortness of breath and cough while improving lung functions. They can also slow disease progression. As an example, smoking cessation refers to quitting smoking cigarettes or other tobacco-related products to slow emphysema progression [77]. Preventive measures comprise avoiding exposure to environmental irritants and pollutants (e.g., outdoor pollution) that can worsen the disease. Please refer to the paragraph on the management and treatment of emphysema for additional information.

### 5.2. Pharmacological Approaches

**Figure 2:**
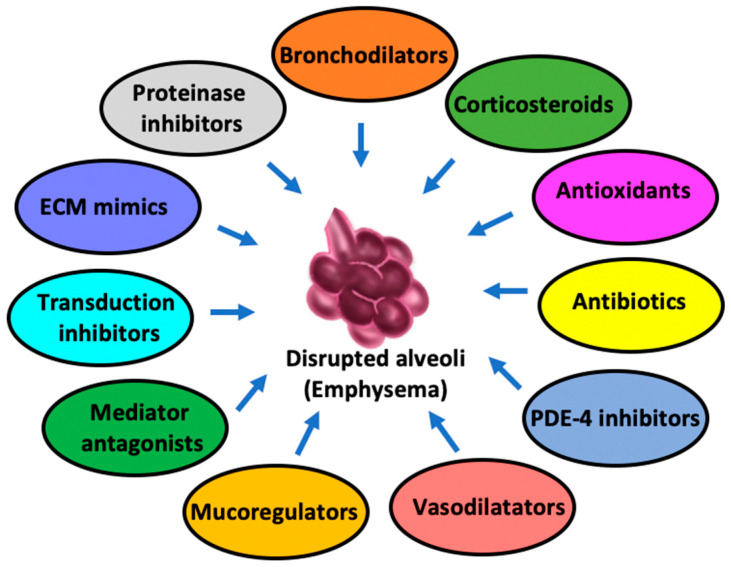
Pharmacologic alternatives against pulmonary emphysema.

Pharmacological approaches primarily focus on improving lung function and preventing its worsening by avoiding exacerbations, not to mention managing symptoms as well. Of note, these approaches are most effective when combined with non-pharmacological strategies. The main categories of treatments concern the following (Figure 2).

#### 5.2.1. Bronchodilators

Bronchodilators are key components in the management of emphysema in that they help alleviate the reduced airflow and breathing difficulty by relaxing the muscles around the airways. There are two main types of bronchodilators: *β*-2 agonists (including short- and long-acting) and anticholinergics.

#### 5.2.2. *β*-2 Agonists

**Figure 3:**
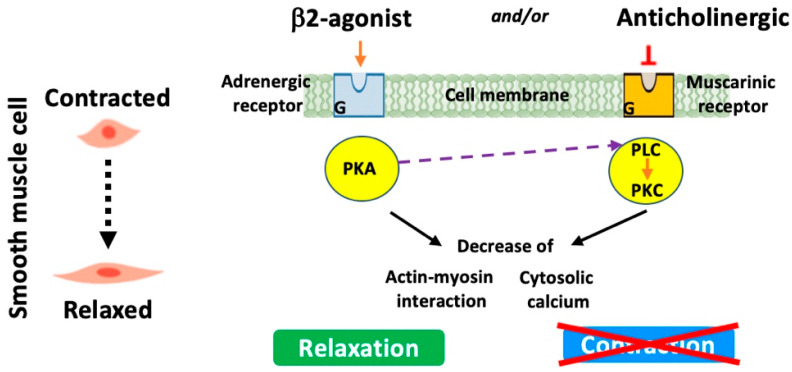
Modes of action of *β*-2 agonists and anticholinergics.

These medications stimulate *β*-2 adrenergic receptors leading to the relaxation of the smooth muscles surrounding the airways (Figure 3). This results in the dilation of the conducting airspaces, making it easier for air to move in and out of the lungs. Briefly, *β*-2 agonists specifically bind to and activate G-protein-coupled receptors that in turn trigger the stimulation/activation of a series of enzymes. These include adenylyl cyclase, which converts ATP to cyclic AMP (cAMP). Increased cAMP will then activate protein kinase A. This latter phosphorylates various targets, resulting in the inhibition of myosin light-chain kinase and reductions in intracellular calcium and hence the relaxation of smooth muscle cells and airway dilatation. Short-acting *β*-2 agonists such as albuterol or salbutamol and levalbuterol are often used for quick relief from acute symptoms and are commonly referred to as “rescue inhalers”. However, long-acting *β*-2 agonists (LABAs) (e.g., salmeterol and formoterol) provide a more prolonged bronchodilator effect and are often used as maintenance therapy to prevent symptoms [78]. Newly developed LABAs such as indacaterol are now suitable for once-daily administration (Table 4) [79,80,81].

#### 5.2.3. Anticholinergics

These medications work by blocking muscarinic receptors (especially M3 receptors) in the airway smooth muscle and submucosal glands and therefore the action of acetylcholine, a neurotransmitter that causes smooth muscle contraction (Figure 3). By inhibiting this action, anticholinergics help open up the airways, making breathing easier. Short-acting anticholinergics like Ipratropium bromide (Atrovent) help in blocking, for short periods, the action of acetylcholine, but long-acting anticholinergics (LAMAs), a recommended treatment mainstay, sustain the relief and are used for maintenance therapy. Other long-acting anticholinergics including the anticholinergic glycopyrrolate (whose pharmacological properties resemble tiotropium bromide) and aclidinium bromide (LAS34273) have recently been developed (Table 5) [81,82,83,84,85,86].

Of interest, additive effects of anticholinergics particularly short-acting ones and *β*-2 agonists have led to the development of combination inhalers [87]. Also, it has been reported that LABAs and tiotropium may also have additive effects, suggesting that a once-daily inhalation of these types of combinations might suffice for their efficiency. Lastly, both LABAs and LAMAs have been reported to exhibit other properties such as anti-inflammatory action and reducing exacerbation frequency [88].

#### 5.2.4. Methylxanthines

The representative medication, theophylline, acts by relaxing the smooth muscles in the airways and has a bronchodilator effect. However, it is less commonly used today due to potential side effects and drug interactions. Recent findings have reported that when theophylline is used at low concentrations, it has beneficial anti-inflammatory effects [89]. More interestingly, this molecule appears to have the potential to ‘‘unlock’’ the resistance to corticosteroids, frequently seen in emphysema patients. This observation has launched research for the design of novel anti-inflammatory theophylline-like molecules that could be used alone or in combination with corticosteroids, unlocking, again, the steroid resistance that limits its clinical usefulness in emphysema treatment. Among commercially available theophylline-like molecules is Doxofylline (Doxofyl), a newer xanthine derivative with bronchodilator properties similar to theophylline but with fewer side effects.

The choice of a bronchodilator depends on various factors, including the severity and frequency of symptoms, and individual patient characteristics. Different types of bronchodilators could be combined or used with other anti-inflammatory medications for optimal management (e.g., with inhaled corticosteroids, see below). It must be emphasized that while these medications can provide relief from symptoms, they do not cure emphysema. This is why they are often used as part of a comprehensive treatment plan that could involve, for instance, smoking cessation and lifestyle modifications (e.g., diet, activities).

#### 5.2.5. Corticosteroids

**Figure 4:**
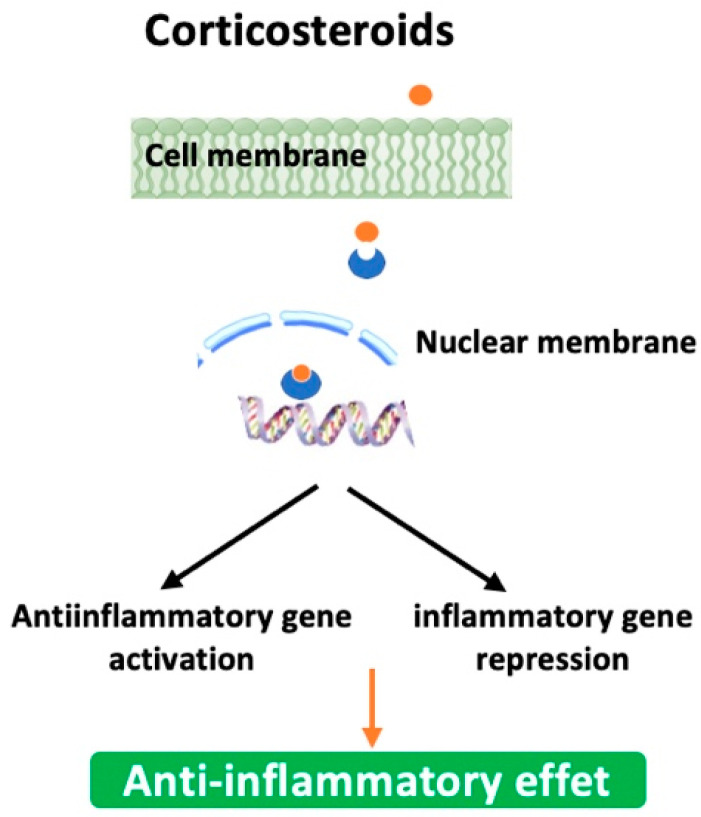
Mode of action of corticosteroids.

Also known as steroids, they work by reducing inflammation in the airways, which can help improve breathing and reduce symptoms. Briefly, they first bind to glucocorticoid receptors in the cytoplasm of targeted cells, particularly the inflammatory cells, macrophages, neutrophils, and T-cells. Next, the steroid-receptor complex moves into the nucleus where it activates anti-inflammatory genes and represses pro-inflammatory genes by down-regulating their expression or inhibiting their transcription factors like NF-κB and AP-1 (Figure 4). It must be emphasized that their clinical use is especially reserved to patients experiencing frequent exacerbations. Moreover, corticosteroids could be used in combination with long-acting *β*-2 agonists and/or anticholinergics (e.g., Symbicort (Budesonide + Formoterol (LABA)); Trelegy Ellipta (Fluticasone + Vilanterol (LABA) + Umeclidinium (LAMA)). Table 6 summarizes some of the corticosteroids explored against emphysema with improved efficacy and reduced side effects [90,91,92,93,94]. They can be delivered in several different ways, including inhalers, pills, and injections, although inhalation is the most common way to deliver this medication for emphysema. However, they may not be effective for every patient as they may increase the risk of infection or lead to weight gain, high blood pressure, and osteoporosis. Another inherent factor to the inefficacy of corticotherapy is corticosteroid resistance [95]. This is why their use, dosage, and treatment period are usually individualized for each person based on their medical history and/or specific needs [96].

#### 5.2.6. Antibiotics

Although antibiotics do not represent a treatment strategy for emphysema (the disease is not caused by an infection), their use is beneficial to patients prone to or experiencing bacterial-infection-mediated acute exacerbations [97]. Indeed, it has been reported that antibiotic therapy results in a more rapid recovery from exacerbations than placebo. Another relevant aspect of antibiotics, especially with the macrolide family, involves the anti-inflammatory properties of these latter in addition to their antibiotic function. At least in vitro, macrolide antibiotics exhibit anti-inflammatory effects including the inhibition of reactive oxygen species generation, inhibition of chemotaxis, and inhibition of cytokine expression [98]. This suggests that their benefit might be more than just an anti-bacterial effect. Clinical trials have been carried out investigating the potential benefit in patients with emphysema. Table 7 depicts a selection of recently developed antibiotics that might be effective in the treatment of episodic bacterial exacerbations of emphysema [99,100,101].

#### 5.2.7. Phosphodiesterase-4 Inhibitors

Among the phosphodiesterases (PDEs), PDE4 is predominantly expressed in neutrophils, CD4+, CD8+ cells, and monocytes/macrophages [102]. Also, PDE4 is expressed in airway smooth muscle and epithelial cells. Accordingly, PDE4 has been reported to be involved in inflammation and airway constriction, making it an interesting target to treat emphysema by improving lung function [103]. Typically, they are taken orally in combination with bronchodilators and/or inhaled corticosteroids. Selective PDE4 inhibitors include rolipram, roflumilast, and cilomilast with adverse side effects such as nausea, diarrhea, headache, and insomnia. To overcome these side effects, other PDEs have been investigated and inhibitors against them, developed preserving the bronchodilatory and anti-inflammatory effects while having less propensity to side effects, are now in clinical development. These include PDE3/PDE4 dual inhibitors like Ensifentrine (RPL554) that display both bronchodilatory and anti-inflammatory benefits [104]. PDE5 inhibitors (e.g., sildenafil) may benefit a subset of patients with pulmonary hypertension or significant vascular remodeling.

#### 5.2.8. Mediator Antagonists

##### Lipid Mediator Inhibitors

Among the lipid mediators that are increased in emphysema are prostaglandin (PG) E2, thromboxane, and leukotrienes [105]. Within these latter, cysteinyl leukotrienes (cys-LTs) are not increased in the exhaled breath of patients as they are in asthma and in principle, these is no need of use of their receptor antagonists, such as montelukast, in emphysema patients. But, targeting leukotrienes has proven to be a disappointing approach because of the redundancy in these mediators. And, it seems that these antagonists would be more effective in patients with emphysema who have a history of allergies or asthma [106].

##### Leukotriene B4 Inhibitors

Much attention has focused on leukotriene B4 (LTB4) because of its property of attracting and activating neutrophils by way of high-affinity interaction with its receptors on the cell surface. In a study, LTB4 also showed potent immunomodulatory effects and had a potent chemotactic activity for CD8+ T-cells via its receptors [107]. LTB4 concentrations were found markedly increased in the sputum or exhaled breath of patients, especially during exacerbations [108]. A series of LTB4 receptor antagonists were (and continue to be) developed for the treatment of neutrophilic inflammation because of their potential clinical value in emphysema treatment, especially during periodic exacerbation phases (e.g., BIIL-284) [109]. But clinical trials have failed to prove their efficiency. As LTB4 is synthesized by 5′-lipoxygenase, attempts have been made to inhibit this enzyme (e.g., GSK2190915, Zileuton) but have also failed because of side effects including hepatic toxicity [110].

##### Tumor Necrosis Factor-α Inhibitors

TNF-α and soluble TNF receptor levels are increased in the sputa of emphysema patients [111]. Given the pleiotropic role of TNF-α in terms of inflammation (e.g., TNF-α induces IL-8 and other inflammatory mediators in airway cells via the activation of NF-kB, thereby amplifying inflammation), it has been hypothesized that monoclonal TNF antibodies (e.g., infliximab, etanercept, golimumab) may be effective in COPD and thus emphysema [112]. Unfortunately, trials of anti-TNF therapies in patients with systemic features of COPD have been inconclusive [113]. Of interest, TNF-α-converting enzyme (TACE), which releases soluble TNF-α, and p38 mitogen-activated protein kinase (MAPK) inhibitors represent potentially attractive targets. Studies are warranted to determine the relevance of such a hypothesis.

##### Other Cytokine Targets

Based on their inflammatory characteristics, other cytokine could potentially be targets in emphysema. These include IL-1β, which has similar amplifying effects to TNF-α; IL-6, whose concentrations are increased in emphysema patients and/or during exacerbations; IL-11; and IL-17. Therefore, inhibitors or blocking antibodies to these mediators represent therapeutic alternatives that are still under investigation [114].

##### Chemokine Inhibitors

Various chemokines are known chemotactic factors for the recruitment of neutrophils, monocytes, and T-cells into the lungs of emphysema patients. They interact with their G-protein-coupled receptors and are targets for small-molecule inhibitors [115]. For instance, IL-8 is a CXC chemokine markedly elevated in the sputa of emphysema patients in their early disease development and is correlated with disease severity [116]. Blocking antibodies to IL-8 (e.g., monoclonal antibodies ABX-Il8 and MDX-018) and related chemokines have been developed but clinical preliminary data were not promising [117].

Other therapeutic targets could be G-protein-coupled receptors (CXCR). Those that are targeted by the mediators IL-8 and growth-related oncoprotein-a (GRO-alpha) are ideal targets such as CXCR1, linked to cell activation and degranulation, and the high-affinity receptor (CXCR2), important in the chemotactic response. In fact, CXCR2 receptors are expressed on neutrophils and monocytes and the blocking of monocyte chemotaxis may prevent the marked increase in the numbers of macrophages found in the lungs of patients with emphysema [118]. Such a strategy may also be useful in exacerbation episodes where CXCR2 are up-regulated in the airways and in mucus hypersecretion [115,118]. Therefore, strategic targeting using the CXCR2 antagonist could be more useful than that using a CXCR1 antagonist. In this regard, small-molecule inhibitors of CXCR2 (e.g., AZD-5069, SCH527123) have entered clinical trials [119]. Lastly, CC chemokines like monocyte chemotactic protein (MCP)-1, and its receptor, CCR2 (expressed on macrophages and epithelial cells from emphysema patients), could be of therapeutic interest [120]. Indeed, several small molecule CCR2 antagonists (e.g., AZD-4791 and RS504393) are already in clinical development.

#### 5.2.9. Antioxidants

Oxidative stress is a documented mechanism that contributes to the development of emphysema, particularly during exacerbations, as it damages lung cells, leading subsequently to tissue injury and loss of function [121]. This suggests that an antioxidative system could be used as a therapeutic alternative against this pathology. A systematic review of studies with oral N-acetyl cysteine (NAC) in emphysema provided evidence of a reduction in exacerbations [122]. Effective antioxidants, including stable glutathione compounds, analogues of superoxide dismutase, and selenium-based drugs, have been developed for clinical use [123]. Another drug of interest is resveratrol, a phenolic component of red wine. It has marked anti-inflammatory and antioxidant properties [124].

Of relevance, increased NO release (as reflected by the increased activity of inducible NO synthase (iNOS)) in combination with oxidative stress results in the formation of peroxynitrite, a potent radical that nitrates proteins among other molecules and alters their function [125]. Peroxynitrite may also lead to corticosteroid resistance in emphysema [94]. A series of selective inhibitors of iNOS have been developed and in a study, one of these, the pro-drug of L-N6-(1-imminoethyl)lysine gave a lasting reduction in NO concentrations in exhaled breath [126], suggesting that the inhibition of peroxynitrite generation by antioxidants or iNOS inhibitors offers a therapeutic approach in emphysema and may restore corticosteroid responsiveness. Other molecules have been examined for their ability to mitigate oxidative stress and inflammation [127,128,129]. These include Vitamins E and C, known for their antioxidant properties; curcumin, found in turmeric, has anti-inflammatory and antioxidant properties that could benefit emphysema treatment; Coenzyme Q10 (CoQ10) is involved in cellular energy production and has been studied for its potential to reduce oxidative damage; Alpha-lipoic acid helps regenerate other antioxidants; Astaxanthin is a carotenoid with potent antioxidant properties; S-adenosylmethionine has antioxidant and anti-inflammatory properties; L-carnitine is involved in fatty acid metabolism and may have antioxidant properties that could help in managing emphysema.

#### 5.2.10. Signal Transduction Pathway Inhibitors

The activation of inflammatory cells in emphysematous lungs occurs via multiple signal transduction pathways representing, therefore, potential targets for inhibition [130]. Accordingly, several inhibitors of kinases, enzymes involved in these pathways, have been developed and some are in clinical development, but concerns about the specificity and/or safety of this approach have been raised and its efficacy is still questionable. Various signal transduction pathways have been targeted.

##### P38 MAPK Inhibitors

Mitogen-activated protein kinases (MAPKs) play an important role in chronic inflammation and a series of enzyme cascades have now been identified [131]. Among these, the p38 MAPK pathway, activated by cellular stress, regulates the expression of inflammatory mediators including IL-8, TNF-α, and MMPs. Various small-molecule p38 MAPK inhibitors of have been designed, exhibiting a broad range of anti-inflammatory effects [132]. Investigations of some were pursued in clinical trials whereas the development of others was discontinued. For example, AZD7624 aims to reduce exacerbations and inflammation in emphysema patients. Pamapimod (R-1503) suppresses the production of key inflammatory mediators involved in lung tissue damage and inflammation. The development of PF-03715455, which targets inflammation and remodeling processes by reducing the production of cytokines such as IL-1β and IL-8, was discontinued. BMS-582949 was studied in early clinical trials, but the development was discontinued despite the fact that the molecule reduces inflammatory responses and oxidative stress in lung tissues. As a precaution, the inhalation of such drugs may be judicious given the likelihood that these types of “broad-spectrum anti-inflammatory” inhibitors might have some toxicity or impair the host natural immune response.

##### Phosphoinositide 3-Kinase Inhibitors

Phosphoinositide 3-kinases (PI-3Ks) are a family of enzymes that generate lipid second messengers involved in a number of cellular events. Of particular interest, the isoform, PI-3Kc, plays a role in the recruitment and/or activation of neutrophils, monocytes, and T-lymphocytes, which stems the development of selective PI-3Kc inhibitors that could have relevant anti-inflammatory in emphysema [133,134]. As a reminder, PI3K inhibitors are primarily studied in cancer and inflammatory diseases, but their potential in treating emphysema is being explored due to their impact on inflammation and cell survival processes. In this regard, some PI-3K inhibitors might have potential implications for emphysema treatment. Idelalisib (Zydelig), a selective PI3Kδ inhibitor, may impact inflammation pathways relevant to emphysema. Copanlisib (Aliqopa), a pan-class I PI3K inhibitor, could potentially affect inflammation in emphysema. MK-2206, an allosteric inhibitor of AKT, a downstream target of PI3K, might have effects on inflammatory pathways relevant to emphysema.

##### NF-kB Inhibitors

It is largely documented that NF-kB regulates the expression of IL-8 and other chemokines, TNF-α and other inflammatory cytokines, and some MMPs. NF-kB is activated in the epithelial cells and macrophages of emphysema patients, particularly during exacerbations [134]. Therapeutically, various approaches could be explored to inhibit NF-kB and its ensuing abnormal inflammation in diseased states. This includes the development of inhibitors of IkB kinases (IKKs), NF-kB-inducing kinase and IkB ubiquitin ligase, which regulate the activity of NF-kB and inhibit the degradation of IkB [135,136]. A series of inhibitors are in experimental stages and have not yet been approved for clinical use. For example, both curcumin and resveratrol, in addition to their antioxidant properties, have been studied in preclinical models for their ability to inhibit NF-kB activation and reduce inflammation in various diseases including emphysema. Bortezomib, an FDA-approved proteasome inhibitor used primarily in cancer treatment, has demonstrated potential in blocking NF-kB activation and may be explored for emphysema. Ginkgolide B, extracted from Ginkgo biloba, has been shown to inhibit NF-kB and could have potential benefits for inflammatory conditions like emphysema.

#### 5.2.11. Proteinase Inhibitors

A working hypothesis of emphysema pathogenesis is the imbalance between proteinases (that degrade structural proteins particularly elastin) and their physiologic inhibitors (i.e., antiproteinases) that prevent such destruction [137]. This means that therapies aiming at either inhibiting these proteolytic enzymes or modulating concentrations of endogenous physiologic inhibitors (e.g., alpha-1 antitrypsin (AAT), elafin, secretory leukoprotease inhibitor, tissue inhibitor of matrix metalloproteinases (MMPs)) could theoretically prevent the initiation and/or progression of the pathology. Progress has been made in identifying both the enzymes involved in tissue destruction in emphysema and their endogenous inhibitors that counteract this degradation [16].

##### Neutrophil Elastase Inhibitors

Neutrophil elastase (NE) is a serine protease that is specific to neutrophils and has a potent elastolytic activity and can activate other tissue-degrading proteinases. NE is inhibited predominantly by AAT. AAT augmentation is used as a treatment of choice for patients with severe AAT deficiency, suggesting that this approach could be effective in emphysema treatment, especially during exacerbations, where free NE activity is often detected [138]. But controlled trials of intravenous instillation of purified AAT have had disappointingly limited beneficial effects [139]. Technically, AAT could be delivered either in recombinant form or by viral vector gene delivery. These approaches are, however, limited by the fact that large amounts of protein are required and gene therapy is unlikely to ensure sufficient protein amounts. In addition, there are challenging concerns related to the delivery of the inhibitor to defined lung areas, where the proteolytic damage causes emphysema. Despite all these concerns and because NE causes other emphysema clinical impairments (e.g., mucous gland hyperplasia, mucus secretion, impaired ciliary function), its inhibition continues to be a potential therapy in such patients. Accordingly, the clinical development of small-molecule inhibitors of NE or monoclonal antibodies is still pursued. In fact, NE represents a “golden” target because in addition to its tissue-injuring capacities, the protease has the potential to activate other proteases and degrade their corresponding physiologic inhibitors, amplifying, thereby, the proteolytic environment [140]. Of relevance, NE inhibitors target also other serine proteinases released from neutrophils, namely cathepsin G and proteinase-3, that possibly contribute to emphysema development [3,16]. Table 8 shows a list of NE inhibitors that have shown therapeutic potential or are being explored in emphysema treatment [16,141,142,143,144,145].

##### Others Protease Inhibitors

Other proteinases possess elastolytic activity, and hence, a potential deleterious impact on lung homeostasis. These include the cysteine proteinases and MMPs [16]. But their implications in emphysema are still debatable. Yet, inhibitors against cathepsins B, K, S, and L, members of the cysteine proteinase family, which are released from macrophages, have been developed [146]. MMPs with elastolytic activity (e.g., MMP-9 and MMP-12) have also been targeted. Search studies were carried out to identify more selective/specific inhibitors and/or target the delivery to the lung parenchyma. Table 9 shows a list of protein inhibitors that have shown potential or are being explored in emphysema treatment [16,147,148,149,150,151].

It must be emphasized that since many proteinases seem to be involved in emphysema development, targeting a single protease may not suffice to protect the lungs against tissue destruction.

#### 5.2.12. Protective Extracellular Matrix Mimics

The degradation of the extracellular matrix (ECM), including elastic fiber, contributes substantially to airspace enlargement as seen in emphysema. Therefore, preventing or slowing such degradation could be therapeutically judicious. Indeed, supplementing ECM components via inhalation has been shown to protect elastic recoil and lung architecture [152,153,154]. The mechanistic rationale is that these mimics might limit, for instance, elastin degradation by simple binding that prevents tissue-degrading protease access, buffer mechanical stress and inflammation, and modulate cell–matrix signaling. In addition, this prevents tissue degradation; hence, the matrix helps a homogeneous wide spreading of mechanical forces throughout the lung for better lung mechanics (stretch, recoil, tension) and alveolar stability [37,155]. Among these mimics, high-molecular-weight (MW) molecules and fibulin-5 have been reported as promising early-disease stage therapeutics. Caution must be, however, paid to these mimics since some behave as a “double-edged sword”. In fact, low-MW hyaluronan and elastin peptides worsen inflammation while high-MW hyaluronan molecules are protective [156,157]. At any rate, the proof of concept showing that the ECM mimics the therapeutic impact has been reported in recent years through a series of clinical trials. The inhalation of high-MW hyaluronan reduced elastin degradation, improved lung function (improved FEV and dyspnea scores), and mitigated inflammation including during acute exacerbations and in patients with alpha-1 antitrypsin deficiency in a study [158].

#### 5.2.13. Mucoregulators 

Mucus hypersecretion is commonly seen in emphysema patients, particularly cigarette smokers. This could predispose patients to frequent exacerbations that, in turn, will lead to progressive diminished lung function [159]. This suggests that reducing mucus hypersecretion could be therapeutically beneficial.

Different mucolytic molecules have been (and continue to be) developed and/or used to reduce mucus production or its viscosity. To this end, the neutrophil inflammatory response has been identified as one of therapeutic targets [160]. In fact, different neutrophil-derived secretagogues (e.g., NE and the oxidative system) mediate mucus secretion [161]. Proteinase inhibitors and PDE4 inhibitors (developed earlier) might, therefore, be effective in modulating mucus hypersecretion. Among the other therapeutic targets are epidermal growth factor receptors (EGFRs) and calcium-activated chloride channels (CACCs) [161,162]. EGFRs, expressed in goblet cells and submucosal glands, mediate mucus secretory responses to various secretagogues that include reactive oxygen species, cigarette smoke, and inflammatory cytokines. Intuitively, the small-molecule inhibitors of EGFR kinase (e.g., gefitinib and erlotinib) that have been developed for the treatment of non-small-cell lung cancer might be worth exploring in emphysema patients. The other alternative involves the inhibition of CACCs, a channel that has been reported to play an important role in mucus secretion from goblet cells and small-molecule inhibitors of CACCs, by molecules such as niflumic acid and talniflumate, which have been developed and tested in patients of interest [163]. Among the classic mucoregulators are carbocisteine, an established mucolytic agent that reduces the viscosity of mucus and continues to be of interest in new formulations and combinations. N-acetylcysteine (NAC), which has antioxidant properties, helps break down mucus. Research into its long-term benefits and optimal dosing is still pursued. Newer enzyme-based mucolytics such as recombinant enzymes targeting specific components of mucus are under investigation. Combining mucoregulation with anti-inflammatory and/or anti-microbial properties is a novel approach. For example, some research is exploring agents that combine mucolytic effects with antibiotic or anti-inflammatory properties. Emerging biologic treatments targeting specific inflammatory pathways involved in mucus production and clearance are being researched as well.

#### 5.2.14. Pulmonary Vasodilators

Vasodilators work by relaxing the blood vessels, including those of the lungs, which can help improve blood flow and oxygen delivery. Although these molecules are primarily used to treat hypertension, they have also been investigated as a potential treatment for emphysema [164]. The rationale is that the pathology leads to the elevation of pulmonary arterial pressure, which correlates inversely with lung function. This is probably due to remodeling of small pulmonary arteries and destruction of the pulmonary vascular bed associated with hypoxic vasoconstriction. A series of pharmacological pulmonary vasodilators have been proposed and/or explored. These include calcium channel blockers, hydralazine, ACE inhibitors or angiotensin antagonists, and endothelin antagonists (e.g., Macitentan and bosentan) since endothelin (ET)-1 is a potent pulmonary vasoconstrictor whose expression is increased in pulmonary vessels in emphysema patients [165]. Prostacyclin is a potent pulmonary vasodilator and analogues, including treprostinil, beraprost, and ileoprost, could represent an interesting alternative [166]. Also, the selective targeting of prostacyclin receptor (e.g., Selexipag, receptor agonist) is an attractive approach that helps dilate pulmonary and systemic arterial vascular beds, reducing pulmonary artery pressure.

PDE5 inhibitors, such as sildenafil and tadalafil, have been shown to reduce hypoxic pulmonary vasoconstriction and arterial remodeling, suggesting a potential for these molecules in emphysema treatment [167]. But the therapeutic impact of these above-cited targets is yet to be assessed.

## 6. Disease Biomarkers

Biomarkers play a crucial role in understanding, diagnosing, and monitoring disease evolution for instance during treatment. With respect to emphysema, there are established and emerging biomarkers. Some of them are useful for the early detection of disease development and/or for assessing the efficacy of therapeutic treatments in clinical trials, especially when structural changes in the lung precede functional decline. Among these are the soluble receptor for advanced glycation end products (sRAGE). Levels of this blood biomarker have been reported to correlate with disease severity and decline in lung function and are now part of the biomarker qualification program for emphysema assessment [168]. Desmosine, a unique cross-linking amino acid of elastin, emerged as a sensitive and specific biomarker for alveolar wall destruction. Of importance, recent advances in mass spectrometry and ELISA techniques have improved the sensitivity and specificity of desmosine measurements in different fluids such as bronchoalveolar lavage fluids, the sputum, the plasma/serum, and urine. These measurements translate real-time elastin turnover, allowing, as indicated above, an early detection and/or monitoring of therapeutic efficacy of emphysema in both clinical and experimental settings [169].

Other biomarkers associated with emphysema merit consideration and could be organized by category. These involve genetics, the airway epithelium, omics, and inflammation [170,171]. Also, functional tests and imaging, although not “stricto sensu” biomarkers, are used in emphysema characterization. Lastly, among the emerging biomarkers that are still under investigation are mircoARNs known for their roles in inflammation and remodeling processes and extracellular vesicles (especially those encapsulating mircoARNs) considered as signatures for cell injury or stress responses [172,173].

## 7. Management and Treatment of Emphysema

Although this is not the scope of this review, a succinct summary about emphysema management is presented and documented with referenced links for more information on this topic. While there are various guidelines (e.g., ATS/ERS (American Thoracic Society/European Respiratory Society) Clinical Practice Guidelines) for the management of COPD including emphysema, GOLD (Global Initiative for Chronic Obstructive Lung Disease) remains the most widely used guideline with explicit recommendations [4,174,175,176,177]. It underscores the strategies to be pursued for better disease management. Thus, smoking cessation remains the first-line protective measure to slow emphysema progression. The pharmacotherapy involves the use of the bronchodilators LAMAs/LABAs with or without corticotherapy to improve symptoms and the quality of life. The pulmonary rehabilitation also alleviates symptoms improving the quality of life. Computed tomography (CT) imaging is ideal, especially for assessing emphysema severity and determining the requirement (or not) for lung volume reduction. Oxygen therapy is indicated for patients with severe emphysema characterized by an impaired ability of the tissues to take in oxygen (resting hypoxemia). Vaccination (influenza, pneumococcal, COVID-19) is highly recommended to reduce the risk of exacerbations. Alpha-1 antitrypsin deficiency screening is recommended for all patients with emphysema, particularly those under age 45 years or non-smokers. Emerging therapies (still not widely used) include, as mentioned above, ECM-stabilizing agents (e.g., hyaluronan inhalation) and biomarkers for monitoring disease evolution.

With respect to lung volume reduction, surgical and endoscopic treatments represent a cornerstone for the management of advanced emphysema and are recommended in major clinical guidelines such as GOLD 2024. For instance, lung volume reduction surgery allows the removal of diseased and non-functional tissues. Such intervention improves elastic recoil and lung mechanics and hence the quality of life and survival. Contrary to surgical intervention, endoscopic (or bronchoscopic) lung volume reduction is less invasive and refers to the insertion of one-way valves in defined areas of the lung to reduce trapped air and hyperinflation that should improve the exercise capacity and quality of life of the patient. Lastly, lung transplantation is intended to end-stage emphysema patients where all other interventions fail. It concerns patients with severe functional limitation who are subject to frequent exacerbations or respiratory failure.

In light of the available pharmacological and non-pharmacological treatments of emphysema and based on the clinical guidelines, such as those of GOLD, a stepwise flowchart of evidence-based treatment could be tailored to the degree of disease severity, taking into consideration the needs of individualized patients [178]. The objectives of such a flowchart are to optimize lung function, preventing exacerbations and improving the overall quality of life. The following are the major steps, some of which have been implemented based on the patient health status. The first common step of this flow is reducing risk factors. This involves smoking cessation, which is largely documented as the most effective way to slow disease progression. Minimizing exposure to environmental pollutants is of importance as well. Influenza and pneumococcal vaccinations are crucial to prevent respiratory infections that could worsen disease symptoms. To improve disease symptoms, pulmonary rehabilitation involving patient-dependent exercises and breathing techniques plays a vital role. This is not to mention the nutritional support for better overall health. Following the assessment of disease severity (e.g., symptoms, spirometry), the stepwise use of pharmacological treatments, namely bronchodilators, consists of using short-acting bronchodilators to for immediate relief while long-acting bronchodilators are employed as the disease gradually evolves. The introduction of corticosteroids in combination with bronchodilators may be required for patients with frequent exacerbations or severe symptoms. For advanced disease, recommendations involve non-invasive (e.g., oxygen therapy) or invasive (e.g., bronchoscopic lung volume reduction) interventions that should preferably be aligned with patient preferences and goals to ensure treatment adherence and hence its success. In the event of the presence of comorbidities such as cardiovascular disease, anxiety, or depression, they need to be handled appropriately because they could impact emphysema management. Last, a personalized action plan is set up for routine follow-up, disease monitoring, and the prevention of exacerbations.

## 8. Exploratory Approaches

**Figure 5:**
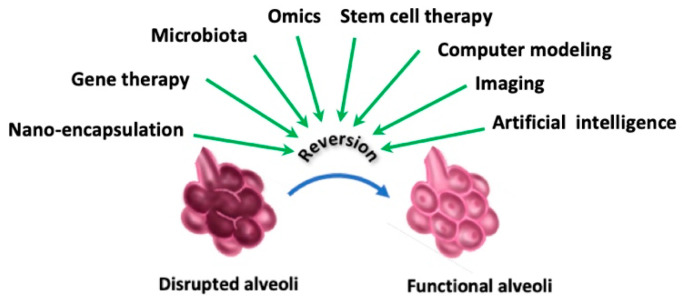
Exploratory approaches to improve lung functions.

Current therapies of emphysema are far from efficient and improved or novel therapies are still needed. That is why more basic and experimental research into cellular, molecular, and genetic abnormalities of emphysema is greatly needed. As such, different research areas are being explored (Figure 5).

### 8.1. Genomics and Proteomics

In this regard, omics studies will allow us to obtain a comprehensive understanding of disease pathogenesis. Genomics refers to the study of the entire genome of an organism, including all of its genes and their functions. Genomics has been increasingly used to study emphysema in order to better understand the underlying genetic factors that contribute to disease initiation and/or progression [179]. In addition to identifying specific genes that are associated with emphysema, genomics can also be used to investigate gene expression patterns in lung tissue from emphysema patients providing mechanistic insights into disease pathogenesis. Consequently, this will allow us to determine, for instance, why only a minority of heavy smokers develop emphysema [180]. Also, genomics may help identify known and unknown genetic targets that are associated with increased susceptibility to cigarette-smoke-induced lung damage or to differentiate disease stages. Moreover, this could help in the design of new molecules to prevent or halt lung damage in emphysema patients. Significantly, this may enable the development of personalized treatments to improve outcomes for emphysema patients. As a powerful example of genomics, distinct patterns of antioxidant gene expression have been identified in airway epithelial cells of smokers [181]. This approach might help identify genes involved in the amplification of the inflammatory process in emphysema, which could help in the discovery of novel drug targets. Another attractive exploratory approach is nutrigenomics. This latter aims to understand the relationship between diet and gene expression and how genetic variations impact nutritional needs and disease risk. With respect to emphysema, as the disease is strongly linked with oxidative damage and chronic inflammation, nutrients such as Vitamins C and E, polyphenols, and omega-3 fatty acids could induce or down-regulate the gene expression of antioxidant defenses (e.g., Nrf2) and inflammation (e.g., NF-κB), respectively. Nutrigenomics might guide antioxidant-rich dietary strategies to mitigate the oxidative stress, an important factor in emphysema development. Last, the dietary components folate, B12, and choline are essential methyl-nutrient donors that might regulate epigenetic processes such as DNA methylation and histone modification, potentially influencing genes involved in lung function and repair in emphysema.

Proteomics could be regarded as a complementary approach to genomics in that it will allow the detection of multiple proteins in lung fluids, tissues and cells [182]. This could help build up patterns of protein expression that relate to specific phenotypes of emphysema where more appropriate targets could be identified, particularly in categorized patients.

### 8.2. Microbiota

‘Microbiota’ describes the living microorganisms found in a defined environment, such as in the lung microbiota. Recent research has shown that the lung microbiota may play a role in the development and progression of emphysema [183]. Studies have found that every emphysema patient has his own (distinct) lung microbiota composition compared to healthy individuals. Also, changes in the lung microbiota are associated with the worsening of emphysema. How the lung microbiota contribute to emphysema is not yet fully understood, but it is believed that chronic inflammation and immune responses to the lung microbiota may play a role in the destruction of lung tissue.

Thus, growing interest/attention has been paid in recent years to targeting the lung microbiota as a potential therapeutic strategy for emphysema. This could be accomplished by using different types of microbiota to promote the growth of beneficial bacteria in the lungs. These include probiotics that have been identified in various studies in terms of their abilities to modulate inflammation and immune function, improving overall respiratory health [184,185]. For instance, *Lactobacillus plantarum* and *Lactobacillus reuteri* have been reported for their anti-inflammatory and immune properties and are being investigated for their effects on lung systemic inflammation [186]. The species *Bifidobacterium lactis*, *Bifidobacterium breve*, *Bifidobacterium longum*, and *Bifidobacterium bifidum* might be beneficial for emphysema management, especially in bacteria-mediated exacerbation phase [187].

Prebiotics are compounds that stimulate the growth or activity of beneficial microorganisms [188]. Among the relevant ones that might be beneficial in emphysema management are inulin, a type of soluble fiber that supports the growth of beneficial bacteria and has been explored for its potential effects on inflammation and immune function. Psyllium Husk and Oligofructose have prebiotic properties and potentially impact systemic inflammation.

Synbiotics are combinations of prebiotics and probiotics designed to enhance the beneficial effects of probiotics by supporting their growth and activity. Research into novel synbiotics for emphysema is still ongoing whereby several combinations are being explored for their potential benefits [189]. Combining, for example, Bifidobacterium longum with oligofructose aims to reduce inflammation, potentially impacting emphysema symptoms.

Fecal Microbiota Transplantation (FMT) is a treatment that involves transferring fecal microbiota from a healthy donor into a recipient’s gastrointestinal tract to restore a balanced microbiome. While FMT is well-established for conditions like Clostridium difficile infection, its application in treating emphysema is still in its infancy. Research into FMT for emphysema is still experimental and protocols or formulations are not yet standardized [190]. Some approaches and considerations related to FMT in the context of emphysema merit consideration. With respect to FMT and the gut–lung axis, investigating how restoring a healthy gut microbiota through FMT might affect the gut–lung axis, potentially influencing lung inflammation and overall emphysema symptoms, is an important alternative to pursue.

The role of the lung microbiota in emphysema is not fully understood. This is clearly an emerging area of research aiming at influencing the gut microbiome to potentially reduce inflammation, therefore improving respiratory health. More research studies are still warranted prior to develop effective microbiota-based therapies.

### 8.3. Micro- and Nanoparticle-Mediated Encapsulation of Drugs

Encapsulation-mediated drug delivery is an attractive approach for the treatment of emphysema. Such an approach relies on the use of particles ranging from micro- to nanometers in terms of size. These small particles can be customized to carry molecules of interest to defined tissues or cells in the body. With respect to emphysema, they can be designed to deliver drugs directly to specified lung areas with the goal to control unwanted inflammatory processes [191].

One approach is to encapsulate molecules within particles with specific properties such as size and surface charge. This will improve their delivery to the lungs by allowing them to penetrate tissues and/or target specific cell types. Moreover, this technology can also allow the drugs to be released slowly over time, which can improve their efficacy and reduce administration and frequencies.

Different types of particles have been investigated for the treatment of emphysema including liposomes, polymeric nanoparticles, and dendrimers (Table 10) [192,193,194,195,196,197,198,199,200,201,202]. They can be loaded with anti-inflammatory molecules, antioxidants, and protease inhibitors [203,204]. Encouraging animal experimental data have shown that encapsulation-mediated delivery enhances the therapeutic efficacy of molecules of interest against emphysema, suggesting the possible applicability of such approach in human patients [205]. Readers must be, however, reminded that issues related to the manufacturing of particles, their stability, and their safety, especially in humans, need to be examined with caution. Table 11 lists some examples of innovative nanoparticle-encapsulated drugs that have shown promise or are under investigation for emphysema management [206,207,208,209,210].

### 8.4. Stem Cell Therapy

In recent years, stem cell therapy has emerged as an exciting research field that showed potential applications in the treatment of lung diseases [211,212]. While there are challenges to overcome, regenerative medicine offers hope for new and effective therapies, especially that ongoing research and clinical trials advance our understanding of biological and cellular processes and capabilities. The application of stem cell therapy to emphysema aims to help repair damaged lung tissue, modulate the immune response, and reduce inflammation to overcome long-term breathing problems and poor airflow. Emphysema is also characterized by fibrosis of small airways and stem cell therapy could stop this phenotype while promoting tissue regeneration. Various stem cells could be used [213]. For example, Mesenchymal Stem Cells (MSCs), derived from bone marrow, adipose tissue, or umbilical cord blood, can differentiate into lung cells [214]. Induced Pluripotent Stem Cells (iPSCs), obtained from adult cells reprogrammed to a pluripotent state, have the capacity to differentiate into specific lung cell types for personalized medicine [215]. Hematopoietic Stem Cells (HSCs) [216], originally found in the bone marrow and involved in forming blood cells, can also contribute to tissue repair and regeneration. They could be used in combination with other stem cell types. The pluripotent cells, embryonic Stem Cells (ESCs), are derived from early-stage embryos and have the potential to differentiate into any cell type, including those of the lungs [217]. Although, their use is more controversial due to ethical concerns, they are still a subject of research. Airway basal stem cells are naturally found in the airways and have the potential to regenerate airway epithelial cells. Alveolar progenitor cells are studied for their potential to restore normal alveolar structure and lung functions. Lastly, endothelial progenitor cells (EPCs) help form new blood vessels and might assist in repairing damaged lung vasculature in emphysema [218].

Among the characteristic features of these stem cells are their anti-inflammatory, immunomodulatory and regenerative properties. There are, however, some challenges to be considered. The delivery method is crucial to ensure efficient targeting of stem cells and this could be carried out by intravenous or intratracheal instillation. It must be emphasized that risks of immune rejection or potential tumor formation may not be excluded.

### 8.5. Gene Therapy

Gene therapy represents a cutting-edge approach and a promising avenue to treat lung diseases by directly addressing genetic causes [219]. Different approaches could be envisioned to reach this goal. Gene replacement refers to introducing a “healthy” copy of a gene to replace a defective or missing gene. In gene editing approach, genetic mutations are directly corrected using technologies like CRISPR-Cas9, allowing the accurate modification of disease-causing mutations. RNA interference (RNAi) or antisense oligonucleotides could be used to silence identified genes known for their deleterious impact, an approach known as gene silencing. Gene augmentation implies to enhance the expression of protective genes to counteract disease initiation and/or progression.

With respect to emphysema, possible applications could involve enhancing the protective genes of specific inflammatory pathways, oxidative stress, and tissue damage, resulting in reducing inflammation and promoting repair. As with stem cell therapy, there are also challenges that merit careful attention with gene therapy [219]. The delivery method relies on the commonly used vectors, adeno-associated viruses (AAVs), and lentiviruses. Other non-viral methods include liposomes and nanoparticles to deliver genetic material or electroporation that temporarily render cells permeable to DNA. Some issues must be considered with this technology. These concern delivery efficiency by overcoming physical barriers like mucus and the immune response and ensuring that the genetic material is taken up by targeted lung cells. Also, one must consider whether the therapeutic effect is sustained over time without any undesired immune responses or disruption of the expression of other genes. Lastly, gene therapy offers other avenues in that it could be combined with other therapies such as medicinal treatment or stem cell therapy or tailored to individual genetic profiles for more effective treatment (personalized medicine). Different gene therapy strategies are currently being investigated. With respect to alpha-1 antitrypsin deficiency, whose patients are at high risk of developing emphysema, introducing a normal copy of its corresponding gene known as SERPINA1 that carries several mutations might lead to the expression of functional alpha-1 antitrypsin to protect lung tissue against neutrophil serine proteinases, especially neutrophil elastase [220]. Gene therapy approaches aiming to deliver SOD2 genes or enhancing Nrf2 activity to reduce oxidative stress and damage in the lungs constitute another alternative. Bcl-2 is an anti-apoptotic protein. Gene therapies that deliver Bcl-2 can potentially prevent the excessive cell death that contributes to tissue damage. In telomerase gene therapy, the enzyme can potentially counteract cellular aging and senescence in lung cells, which may be relevant given the age-related nature of the disease [221]. TGF-β is involved in airway remodeling and gene therapies targeting TGF-β signaling could help in modulating fibrosis and airway changes [222]. To reduce inflammation and tissue damage, gene therapy could involve delivering anti-inflammatory mediator IL-10 gene or gene that encode TNF-α inhibitor to the lungs [223].

### 8.6. Computer Modeling

This approach offers several advantages. With respect to emphysema, it allows the simulation of the gradual destruction of alveolar structures, the contribution of the mechanical forces to tissue dysfunction, and the potential impact of therapeutic applications. According to the literature, there are different types of computational models: a model that simulate elastic network (2D or 3D) that mimics alveolar walls of lung parenchyma; another model that reproduces local mechanical responses in response, for instance, to breathing or coughing; a model that replicates cellular behavior following stimulation (e.g., response of epithelial cells to endogenous or exogenous stimuli); and multiscale models that integrate the properties of the models, thereby including molecular, cellular, tissue, and organ-level processes. The key feature of these models is that they contribute to decode the disease dynamics that are crucial for the design of tailored therapeutic strategies [224,225].

### 8.7. Imaging Techniques

Magnetic resonance imaging (MRI), although of limited use in routine clinical practice due to lower sensitivity compared to computed tomography (CT) scans, can provide functional information about lung perfusion and ventilation, not to mention the advantage of absence of ionizing radiation. Recently, Overhauser Magnetic Resonance Imaging (OMRI) has emerged as a type of magnetic resonance imaging (MRI) technique that enhances the sensitivity of the image. Because OMRI can provide detailed information on lung morphology and function, it has been proposed as a potential tool for detecting and/or monitoring pulmonary emphysema evolution particularly in the treatment phase [226]. In fact, one of the advantages of OMRI is that it can detect changes in lung function and structure at an early stage before symptoms become clinically apparent. OMRI can also differentiate between different types of lung tissue, such as healthy lung tissue and emphysematous tissue, which can be difficult to distinguish with other imaging techniques. However, OMRI is still a relatively new technology and more research is needed to fully evaluate its diagnostic and monitoring potential in emphysema and other lung diseases.

### 8.8. Artificial Intelligence

Although artificial intelligence (AI) is not considered as a therapeutic device, it is clearly and increasingly being utilized in the diagnosis, management, and treatment of diseases including emphysema [227].

With respect to diagnosis, AI can analyze CT scans to detect and quantify emphysema [228]. It can identify patterns of lung tissue damage with high precision, which is indicative of emphysema. Significantly, radiomics, an approach that comprises a large number of features from medical images using data-characterization algorithms, uses AI to extract a large number of quantitative features from medical images, providing a detailed characterization of lung tissue that goes beyond what is visible to the naked eye [229,230].

Regarding the management, from a prognostic standpoint, AI can, for instance, analyze real-time data about respiratory function and symptoms collected from patients with wearable devices and mobile health applications. As such and when appropriate, alerts for early intervention will be generated, which could limit hospital visits and improve overall disease management. Also, AI can assess the risk of acute exacerbations in patients, allowing for preemptive measures to avoid hospitalizations [231]. More interesting, AI can predict disease progression by analyzing patient data that combine medical history, genetic information, and living habits [232]. This represents an important opportunity for clinicians to tailor management plans for individual patients with the goal of potentially improving outcomes.

Therapeutically, to prevent medical error or at least reduce side effects often seen in chronic disease management, AI can help develop personalized treatment strategies (e.g., medication combinations and dosages) by comparing large datasets to identify treatment that best fit patients based on their specific profiles [233]. To manage emphysema effectively, AI can also guide patients in their pulmonary rehabilitation exercises, offer them motivational support, and monitor their adherence.

Last but not least, AI could accelerate the development of new therapies by predicting how new compounds will interact with biological targets involved in emphysema. Furthermore, AI can analyze clinical trial data to eventually identify biomarkers for response to treatments [234]. Of importance as well, AI should be able to analyze genetic data to pinpoint genetic predispositions to emphysema, hence contributing to a better understanding of its pathogenesis and identifying potential targets for gene therapy [235,236].

## 9. Conclusions

Emphysema is a progressive, destructive, and irreversible lung inflammatory disease. Cigarette smoking represents the most frequent etiologic factor of disease pathogenesis. With the exception of bronchodilators, there are no efficient therapies to halt or at least slow down the course of this pathology. This suggests that the disease pathogenesis is still incompletely understood, suggesting that either the already explored therapeutics need to be “revisited” or novel targets need to be identified. Indeed, innovative therapeutic strategies are being explored with the perspective of personalized or evidence-based medicine. With respect to personalized medicine, it must be emphasized that the treatment of emphysema should no longer rely on the classical taxonomic/descriptive classifications of COPD that categorizes this latter into fixed subsets. Indeed, such an approach disregards the diversity of the underlying disease mechanisms, clinical heterogeneity, and dynamic progression of each disease subset. Rather, management should be personalized guided by molecular biomarkers alongside imaging patterns and biological drivers in order to enable the use of precision therapies. Importantly, therapeutic strategies should target not only the source of initiation and/or progression of the disease but also self-amplifying mechanisms that sustain emphysema themselves, namely airspace enlargement, ECM breakdown, and progressive mechanical failure, all of which should hinder the self-perpetuation of tissue damage and stabilize lung architecture.

## Figures and Tables

**Table 1:** Risk factors precipitating emphysema development.

Risk Factor	References		References		References
Smoking Passive smoking	[4] [5,6]	Air pollution Industrial fumes	[6,10] [6,7,11]	Occupational exposures Dust inhalation at work	[7] [6]
Alpha-1 antitrypsin deficiency Genetics	[4,12] [4,9,12]	Age Lung development	[8] [8]	Asthma Repeated respiratory infections	[13] [14]

**Table 2:** Clinically relevant experimental animal models of emphysema.

Trigger	Method	Advantages	Disadvantages	References
Cigarette smoke	Whole body exposure, nose exposure, or intranasal/intratracheal instillation of cigarette smoke extract	Straightforward manipulation Tissue injury in short period of time (several weeks to few months)	Does not fully mimic human emphysema. Short period of exposure (days) versus chronic exposure (years in humans)	[41,42,43,44]
Elastase (e.g., papain, porcine pancreatic elastase, neutrophil elastase)	Intranasal/intratracheal instillation of purified protease(s)	Single instillation-mediated tissue injury Less costly	Does not fully mimic human emphysema. Acute injury that does not translate chronic injury seen in emphysema	[45,46,47]
Chemicals (e.g., ozone, nitrogen dioxide, or sulfur dioxide)	Intranasal/intratracheal instillation of purified chemicals	Effects of specific toxics on the development of emphysema Less costly	Does not fully mimic human emphysema. Acute injury that does not translate chronic injury seen in emphysema	[10,11,48,49,50,51]
Exacerbation (e.g., bacterial infection with *Heamophilus influenzae*)	Intranasal/intratracheal instillation of bacterial strains following chronic exposure to cigarette smoke	Exacerbation of characteristic features of cigarette-smoke-induced emphysema	Does not fully mimic exacerbation phase of human emphysema Does not translate the frequency of episodic phases of emphysema exacerbation	[52,53,54]

**Table 3:** A selection of targeted genes involved in emphysema development.

Targeted Gene											
Enzyme/ Protease	Ref.	Physiologic Inhibitor	Ref.	Chemokines/ Cytokines	Ref.	Receptors	Ref.	Oxidative System	Ref.	Structural Protein	Ref.
NE MMP-12 MMP-9 ADAM17	[19] [35] [60] [61]	AAT TIMP-3	[62] [63]	TNF-a IL-13 IFNg	[64] [65] [66]	TNFR IL-1bR CCR6 TLR4	[67] [67] [68] [69]	Nrf 2 SOD/GPx NADPH oxidase	[27] [70] [71]	Elastin SP-D Fibulin-5	[72] [73] [74]

Abbreviations: NE, neutrophil elastase; MMP, matrix metaloproteinase; ADAM-17, A disintegrin and metalloprotease 17; AAT, alpha-1 antitrypsin; TIMP, tissue inhibitor of metalloproteinase; TNF, tumor necrosis factor; IL-13, Interleukin-13; IFNg, Interferon gamma; TNFR, tumor necrosis factor recemptor; IL-1bR, Interleukin-1b receptor; CCR6, C-C Motif Chemokine Receptor 6; TLR4, toll-like receptor 4; Nrf 2, Nuclear Factor Erythroid 2-Related Factor 2; SOD, superoxide dismutase; GPx, glutathione peroxidase; MPO, myeloperoxidase; SP-D, Surfactant protein D; Ref., references.

**Table 4:** Examples of developed long-acting *β*2-agonists.

*β*2-Adrenergic Agonist	Mechanism	Advantages	References
Indacaterol	Ultra-long-acting agonist with once-daily dosing	Rapid onset of action with sustained bronchodilation	[79]
Olodaterol	Ultra-long-acting agonist with once-daily dosing	Rapid onset of action with sustained bronchodilation; often combined with other bronchodilators	[80]
Vilanterol	Long-acting agonist, combined with inhaled corticosteroids or long-acting muscarinic antagonists	Once-daily dosing with a rapid onset and sustained effect	[81]

**Table 5:** Examples of developed long-acting anticholinergics.

Muscaniric Antagonist	Mechanism	Advantages	References
Tiotropium	Prolonged bronchodilation	Long-acting duration of effect (once-daily dosing); lower risk of systemic side effects	[81,82]
Umeclidinium	Specific reduction smooth muscle contraction in the lungs	Long-lasting effect (once-daily dosing; effective use in dual or triple inhaler therapy (e.g., with LABAs and inhaled corticosteroids))	[83]
Aclidinium	Bronchodilation	Twice-daily dosing; but reduced systemic side effects	[84]
Glycopyrrolate	Reduced bronchoconstriction	Once-daily dosing; highly selective in the lungs; minimal side effects	[85]
Revefenacin	Reduced bronchoconstriction	Once-daily use; minimal anticholinergic side effects	[86]

**Table 6:** Corticosteroids explored against emphysema.

Corticosteroids	Mechanism	Advantages	References
Fluticasone Furoate	Longer duration of anti-inflammatory action compared to fluticasone propionate	Once-daily dosing; lower systemic absorption reducing side effects	[90,91]
Mometasone Furoate	Originally for asthma, it has been explored for emphysema treatment	Once-daily dosing; potent at lower doses; reduced risk of systemic side effects	[92]
*Qvar* (Extrafine Particles)	Reformulated version of beclomethasone	Deeper reach into the lungs	[93]
Ciclesonide	Prodrug activated in the lungs that has been explored for emphysema treatment	Minimal systemic exposure and reduced side effects	[94]

**Table 7:** A selection of antibiotics with potential benefits against exacerbations.

Antibiotic (Class)	Mechanism	Advantages	References
Delafloxacin (Fluoroquinolone)	Inhibits bacterial growth	Broad-spectrum activity; treatment of acute bacterial exacerbations; useful against multidrug-resistant strains	[99]
Lefamulin (Pleuromutilin)	Bactericidal activity	Covers pathogens implicated in exacerbation (e.g., *Haemophilus influenzae*); effective against atypical bacteria and multidrug-resistant strains	[100]
Relebactam (β-lactamase inhibitor)	Bactericidal activity	Useful in severe exacerbations caused by multidrug-resistant Gram-negative bacteria	[101]

**Table 8:** A selection of NE inhibitors aimed to treat emphysema.

NE Inhibitor	Purpose	References
Sivelestat (ONO-5046)	Selective and specific inhibitor of NE; reduced inflammation and tissue damage	[141]
AZD9668	Selective neutrophil elastase inhibitor; treatment of emphysema	[142]
Alpha-1 Antitrypsin Enhancers	Research in AAT efficacy continues for emphysema treatment	[143]
Elafin	Small protein with inhibitory activity	[16]
Neutrophil Elastase Inhibitor Peptides	Specifically designed to target NE	[144]
Biological Agents	Target NE pathway	[145]

**Table 9:** A selection of other proteinase inhibitors aimed at treating emphysema.

Protease Inhibitor	Purpose	References
Marimastat	Broad-spectrum MMP inhibitor; reduced matrix degradation and inflammation	[147]
Batimastat	Broad-spectrum MMP inhibitor; modulation of tissue remodeling	[148]
AZD1236	Selective targeting of MMP-12	[149]
Cathepsin S Inhibitor	Reduced inflammation and tissue remodeling	[16]
Cathepsin K Inhibitor	A selective inhibitor, reduced lung tissue damage and inflammation.	[150]
E-64 Inhibitor	Broad-spectrum cysteine protease inhibitor	[151]

MMP, matrix metalloproteinase.

**Table 10:** List of carriers for drug delivery to the lungs.

Carrier	Type	Size (Diameter)	Purpose	References
Microparticles	PLGA, DPI,	1–5 µm	Controlled release, deposition in the bronchi and alveoli	[192,193]
Nanoparticles	Polymers, lipids	10–500 nm	Controlled drug release, deposition in the alveoli	[194]
Liposomes	Uni- or multi-lamellar vesicles	≥100 nm	Deeper penetration	[195]
Dendrimers	PAMAM	10–100 nm	Penetration through biological barriers, specific lung tissue targeting	[196]
Inorganic nanoparticles	Gold, silica, magnetic	10–100 nm	Imaging and drug delivery to specific lung regions	[197]
Micelles	Polymers, Oil-in-water or water-in-oil	10–100 nm	Better distribution in lung tissue	[198]
Hydrogels	Chitosan, alginate, synthetic polymers	100 nm to few µm	Sustained release, tunable degradation, and drug release profiles	[199]
Nanosuspensions	Pure drug particles	100 to 1000 nm	Improved solubility and lung retention	[200]
Protein-Based Carriers	Albumin or Silk Fibroin Nanoparticles	50 to 500 nm	Biocompatible, biodegradable, and suitable for deep lung delivery and prolonged retention	[201]
Exosomes	Small extracellular vesicles naturally derived from cells	30 to 150 nm	Targeting specific lung cells	[202]

**Table 11:** Examples of nanoparticle-encapsulated drugs in use or under investigation for lung treatment.

Encapsulated Drugs	Formulation	Advantages	References
Resveratrol	PGA-co-PDL or solid lipid nanoparticles	Pulmonary delivery; mitigated inflammation and oxidative damage	[206]
Dexamethasone	Polymeric or lipid-based nanoparticles	Enhanced delivery to the lungs; sustained release; reduced side effects	[207]
Salbutamol	PLGA nanoparticles	Improved lung deposition and controlled release	[208]
Tiotropium	Solid lipid nanoparticles	Better deposition in the lungs; prolonged drug release	[209]
Formoterol	Polymer-Based Nanoparticles	Delivery to the lungs; improved therapeutic effect and reduced side effects	[210]

## Data Availability

No new data were created or analyzed in this study.
